# The Hybridization Barrier between Herbaceous *Medicago sativa* and Woody *M. arborea* Is Weakened by Selection of Seed Parents

**DOI:** 10.3390/plants2020343

**Published:** 2013-05-31

**Authors:** Edwin Bingham, David Armour, John Irwin

**Affiliations:** 1 Agronomy Department, University of Wisconsin, Madison, WI 53706, USA; 2 School of Agriculture and Food Science, University of Queensland, Brisbane, Qld 4072, Australia; E-Mails: d.armour@uq.edu.au (D.A.); j.irwin@uq.edu.au (J.I.)

**Keywords:** species, subspecies, interspecific hybrids, genetics, genome, genome shock, hybrid breakdown, plant breeding

## Abstract

*Medicago sativa*, alfalfa or lucerne, and *M. arborea* were considered reproductively isolated until recently. Then, in 2003, an alfalfa genotype was identified that produced a few seeds and progeny with hybrid traits after a large number of pollinations by *M. arborea*. A derivative of this alfalfa genotype also produced a low frequency of progeny with hybrid traits. Thus, the hybridization barrier was weakened by selection of seed parents. Hybrids from both events expressed traits from *M. arborea* and *M. arborea*-specific DNA bands, although more of the *M. sativa* genome was retained, based on the DNA results. Thus, there was chromatin elimination during embryogenesis, resulting in partial hybrids (hereafter hybrids). However, more than 30 hybrids with an array of *M. arborea* traits have been obtained thus far, and research continues on the nature of the hybrids. Traits have been genetically transmitted in crosses, and selected traits are in use for alfalfa breeding. This paper reviews the first hybrids and then focuses on further weakening of the hybridization barrier with the discovery of a more efficient hybridizer derived from crossing *Medicago sativa* subspecies, *sativa*, *coerulea* and *falcata*. This genotype was found to have reproductive abnormalities associated with its complex subspecies origin that are best described as hybrid breakdown. In effect, this subspecies derivative is a bridge-cross parent that consistently produces hybrids. Reproductive abnormalities in the bridge-cross parent are reported and discussed.

## 1. Introduction

*Medicago sativa* (L.), alfalfa or lucerne, is a forage grown around the world. *M. arborea (L.)* is a woody shrub native to the islands and areas around the Mediterranean Sea. Use of *M. arborea* as a browse plant was recorded by the ancient Greeks, and it has been cultivated as a forage plant since Roman times [1]. *Medicago arborea* has many traits of potential use in alfalfa breeding. It can grow to a height of four meters, it is remarkably drought resistant, and it is the longest-lived *Medicago* species. *Medicago arborea* also has large seeds, disease resistance and morphological traits that could restructure alfalfa.

The transfer of germ plasm from relatives of alfalfa has been important in alfalfa research and breeding. Subspecies relatives that freely intercross with alfalfa include *M. sativa* ssp. *sativa* (alfalfa itself), *M. sativa* ssp. *coerulea* and *M. sativa* ssp. *falcata* (hereafter, sativa, coerulea and falcata, respectively). Species that are more difficult to cross with alfalfa, and often require embryo culture, have been reviewed [2]. The list of successful interspecific hybrids with alfalfa does not include *M. arborea.*

Using morphological characters, Small and Jomphe [3] placed *M. sativa* and *M. arborea* into different sections of the genus *Medicago*, namely sections *Medicago* and *Dendrotelis*, respectively. Steele *et al*. [4] summarized several morphological- and molecular-based phylogenetic studies of the genus *Medicago*, allowing them to consider evolution of characters considered taxonomically important, including those used by Small and Jomphe [3]. Based on an analysis of all of the above characteristics, they concluded that *M. arborea* is part of a group with other species in the section *Medicago*, including *M. sativa*. It was suggested that the common ancestor of *M. arborea*, and its woody relatives *M. strasseri* and *M. citrina*, is an herbaceous perennial in the section *Medicago*.

Historically, the hybridization barrier between *M. sativa* and *M. arborea* (both autotetraploids, 2n = 4x = 32) was thought to be complete. Fredrickson and Bolton in 1963 reported *M. arborea* pollen growth into the ovarian cavity of alfalfa, but no hybrids were obtained [5]. In Wisconsin, USA, direct crosses of alfalfa and *M. arborea* in 1970 and 1985 produced no hybrids, and results were not reported. McCoy and Echt, 1993, stated that it was possible to recover hybrids between alfalfa and all other species of the subgenus *Medicago* with the exception of *M. arborea* [6]. Then, in 1996, Nenz *et al.* [7] reported somatic hybridization of the two species, but the somatic hybrids were not fertile. This report rekindled interest in the hybrid in Wisconsin. The new program began screening for alfalfa genotypes that supported degrees of embryo development after hand crossing with *M. arborea* pollen. Eventually, an alfalfa seed parent that produced progeny with hybrid traits was identified in 2003 [8]. Seed from this seed parent was sent to Australia, where the hybridization was repeated [9]. This was the first level of weakening the hybridization barrier. The first hybrids from both programs have most of the alfalfa genome with introgression of parts of the *M. arborea* genome, based on DNA analysis [9,10,11], and contain much new variation for alfalfa breeding. Recently, anthracnose resistance was transferred from *M. arborea* to alfalfa [9], and alfalfa × *M. arborea* derivatives have been used in alfalfa breeding [10]. Also, traits, such as larger seed and vegetative organs, are currently being backcrossed into alfalfa. 

This paper summarizes interspecific crosses of alfalfa × *M. arborea* in Wisconsin, USA, and Queensland, Australia, over 10 years and reports the number of hybrids produced in recent years. This establishes the repeatability of the interspecific cross with selected alfalfa seed parents, especially one identified as a bridge-cross parent. Reproductive characteristics of the bridge-cross parent are reported and discussed. 

## 2. Experimental Section

### 2.1. Plant Materials

Male sterile alfalfa seed parents were used in Queensland, Australia, and Wisconsin, USA, to maximize the pollen parent contribution and minimize the amount of self-seed and the number of progeny to be screened. Alfalfa and *M. arborea* parents are maintained as clones from shoot cuttings rooted in sand. In the years 1998–2002 in Wisconsin, five male sterile alfalfa parents were screened for hybrid production by pollinating at least 200 florets of each seed parent with *M. arborea* pollen each year. However, no hybrids were obtained, and only alfalfa clone 6-4ms, Table 1, was retained for use in the present study. The alfalfa clones used as seed parents from 2003 through 2011 are listed in Table 1. 

**Table 1:** Descriptions of alfalfa male sterile clones used as seed parents.

Male Sterile	Description and Pedigree
6-4ms *	Male sterile plant discovered in cultivar Saranac in 1972.
MBms *	Male sterile plant from cross of cultivars Magnum III × Blaser XL.
WA 2071 *	Male sterile plant from cross of MBms × Blaser XL.
WA 2625 *	Male sterile plant from cross of (MBms × Peruvian) × Sequel.
M8 **	Male sterile plant discovered after crossing *M. sativa* subspecies, as outlined in Figure 1.

* Purple flower color; ** cream flower color.

Alfalfa clone 6-4ms, Table 1, behaves as a cytoplasmic male sterile and has been used in many studies for more than 40 years [12]. It produces a trace amount of self-seed after extensive self-pollination. Clone MBms, Table 1, also behaves as a cytoplasmic male sterile and produces a trace amount of self-seed. It was developed from cultivars grown *circa* 1990. Clone MBms was used as a seed parent *per se* and used to develop the male sterile plants, WA2071 and WA2625, Table 1, used in Queensland, Australia. WA2071 will produce small quantities of self-seed after extensive selfing, whereas WA2625 does not produce self-seed. 

**Figure 1:**
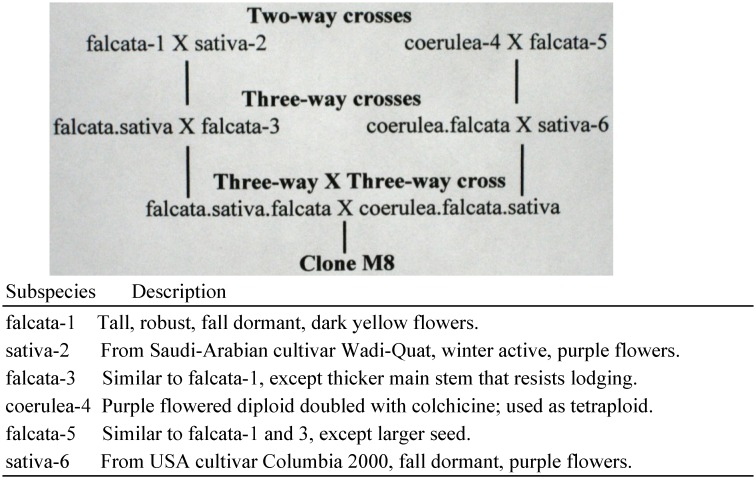
Pedigree of Clone M8.

Clone M8 was first used in 2004 and is still in use. The pedigree of M8 is outlined in Figure 1. Clone M8 has cream colored flowers, due to segregation of genes from purple- and yellow-flowered subspecies parents. Cream colored flowers are useful in a seed parent to distinguish self-progeny with cream flowers from hybrids with yellow flower color. M8 is functionally male sterile, but has a trace of pollen, and produces about five self-seeds per 100 florets self-pollinated. The male sterility of clone M8 is likely due to genetic imbalances associated with its complex parentage. The subspecies parents of M8 all had normal fertility. 

In the first few years that clone M8 was used in crosses, it had an anticlinal, pie-shaped chimera in the ground tissue below the epidermal layer. The pie-shaped chimera was heterozygous for purple flower color and produced gametes with the gene for purple when the chimera was used in the phyllotaxus of node development. Thus, M8 produced an occasional self-progeny with purple flowers, and it has produced three hybrids with variegated flowers. Over years of making shoot cuttings with one node, the chimera has been lost. The chimera did not impact on the reproductive aspects of M8 and is not discussed in the paper.

*Medicago arborea* materials used in Wisconsin [8] were from the Greek Islands and areas around the Mediterranean Sea. No differences as pollen parents in crosses with alfalfa have been detected. *M. arborea* plants used in Queensland traced to the accession SA30528 = CPI131489, collected in Spain [9].

### 2.2. Crossing Procedures

The tools used to transfer pollen from *M. arborea* to alfalfa in Wisconsin changed over the years. From 1998 until about 2007, the tools included the tip of a pocket knife, small stainless steel weighing spatulas and bits of sand paper glued to tooth picks. However, none of these tools catch and retain pollen that becomes airborne when *M. arborea* flowers are tripped onto the tools. With limited *M*. *arborea* pollen most years, a tool was improvised to catch and retain all the pollen. The improvised tool is a piece of plastic soda straw 3–4 cm long attached to a pencil or piece of wooden doweling. Tripping *M. arborea* flowers into the soda straw catches all the pollen and improves crossing efficiency. 

Crossing in Wisconsin was done by hand in the greenhouse, late winter through spring. Crosses were labeled with a small tag attached to racemes with string. Alfalfa racemes typically have 15–20 open flowers at the time of pollination, and the number of flowers crossed was estimated from the number of tagged racemes. *M. arborea* racemes contain fewer flowers, and flowering was sporadic in the greenhouse and limited the supply of pollen most years in Wisconsin. In *M. arborea* × alfalfa crosses, a flowering branch was labeled and dedicated to crosses using alfalfa pollen. In Queensland, *M. arborea* flowered profusely during the winter months, and pollen was always collected on the end of a small stainless steel weighing spatula.

### 2.3. Plant Growth

Seeds of all crosses were scarified by hand using medium grit sandpaper, planted in Jiffy-7 peat moss pellets (Jiffy Products, Norton, MA, USA) and transplanted after 4–5 weeks into pots with Metro-Mix (Sun Gro, Seba Beach, AB TOE 2BO, Canada). In Queensland, UC mix [13] was used (peat:sand, 1:1), fertilized with the necessary nutrients. Plants were grown in naturally illuminated glasshouses in Madison and Brisbane without strict temperature control, over the range 15–35 °C.

### 2.4. Cytology Methods

Chromosome counts were made using root tips 8–10 mm long, collected from potted plants and pretreated in ice water 18–24 hours to shorten the chromosomes and arrest the spindle apparatus. They were then fixed in 3 parts 95% ethyl alcohol, 1 part glacial acetic acid for at least 24 hours before hydrolyzing in 1 part HCl, 1 part 95% ethyl alcohol and smearing the 1 mm growing point in propionic carmine. Pollen also was stained with propionic carmine.

## 3. Results and Discussion

### 3.1. M. arborea × Alfalfa: No Hybrids

**Figure 2:**
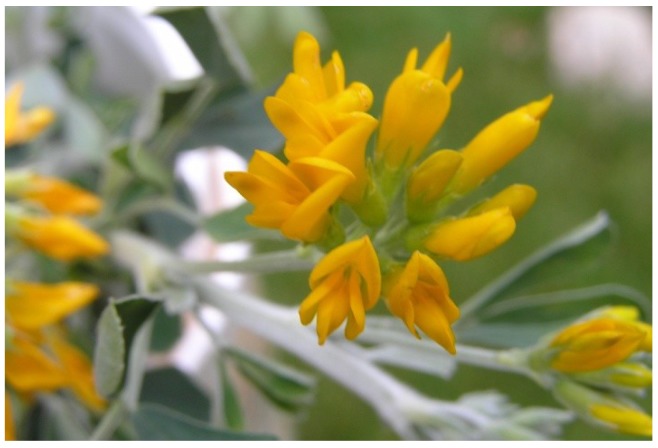
*Medicago arborea* flowers. The figure also shows the maximum number of flowers on a raceme; the raceme has about half as many flowers as alfalfa.

*M. arborea* has yellow flowers (Figure 2), and the yellow pigment is useful in distinguishing hybrids with alfalfa, from self-progeny of *M. arborea*. Four *M. arborea* plants were used as seed parents in *M. arborea* × alfalfa crosses in Wisconsin between 2003 and 2008. At least 100 flowers were crossed each year using pollen from several different alfalfa plants. However, the hybridization barrier using *M. arborea* as the seed parent has been complete. Seed obtained thus far has all been from self-pollination of the *M. arborea* seed parent. Similarly, in Queensland, no hybrids were produced after pollinating hundreds of *M. arborea* flowers over the period 2005–2009. Nonetheless, use of *M. arborea* as the seed parent will continue using hybrid derivatives as pollen parents, with the goal of transferring the subterranean crown of alfalfa to *M. arborea*. 

### 3.2. Alfalfa × M. arborea: The First Sexual Hybrids

In the years 1998–2002 in Wisconsin, five male sterile alfalfa seed parents including 6-4ms were used in crosses with *M. arborea*. No hybrids were produced, and all parents, except 6-4ms, were dropped from the program. Clone 6-4ms was kept in the program, because it carried some pods to maturity. The pods contained minute aborted seeds, and 6-4ms appeared on the verge of producing hybrid seed. Clone 6-4ms has not produced a hybrid over many years of crossing under the same conditions as other seed parents and serves as control with a hybridization barrier.

**Figure 3:**
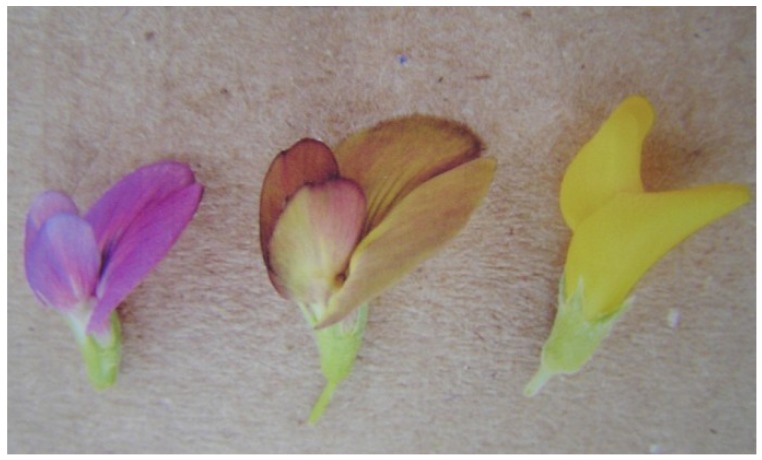
*Medicago sativa*-MBms purple flower, left; hybrid flower, middle; *M. arborea* yellow flower, right. The figure also illustrates coexpression of purple and yellow pigment in variegated hybrid flower, middle; and the large size of hybrid flower, middle.

In 2003, the hybridization barrier was weakened using alfalfa male sterile clone MBms. This parent produced 10 hybrids after *ca* 2,000 flowers pollinated by hand with *M. arborea* pollen, Table 2. Flower color is a distinctive hybrid trait because the purple and yellow pigments of respective parents are coexpressed in the hybrid (Figure 3). Since 2003, *M. arborea* pollen has been in short supply, and MBms has been used in limited crosses. It has only produced one more hybrid, Table 2. 

**Table 2:** Alfalfa plants cross pollinated with *M. arborea* pollen 2003–2011.

Alfalfa seed parents crossed with *M. arborea* (hybrids/florets pollinated)					
	Madison, Wisconsin, USA			Queensland, Australia	
Year	6-4ms	MBms	M8	WA2071	WA2625
**2003**	0/100	10/2,000			
**2004**	0/100	0/300	3/300		
**2006**	0/100	0/100	7/500		
**2007**		0/100	2/250	5/2,100	0/6,000
**2008**	0/100	0/100	2/100		
**2009**			1/150		
**2011**	0/100	1/100	1/50		
**Total**	0/500	11/2,700	16/1350	5/2,100	0/6,000

The hybridization barrier also was weakened in Australia, where five hybrids of WA 2071 were produced after pollinating 2,100 flowers, Table 2. Thus, the interspecific hybridization was repeated in a new environment using a descendant of MBms and different *M. arborea* pollen parents.

In contrast, when WA2625 was used as the male sterile alfalfa female, no hybrids were obtained from over 6,000 florets pollinated, Table 2. The plant had normal female fertility when pollinated with alfalfa pollen, but it had a barrier to interspecific hybridization. The contrasting results between WA2071 and WA2625 indicate a genetic component in the ability of alfalfa plants to hybridize with *M. arborea*.

### 3.3. Medicago sativa Subspecies Derivative M8 × M. arborea: More Hybrids

**Figure 4:**
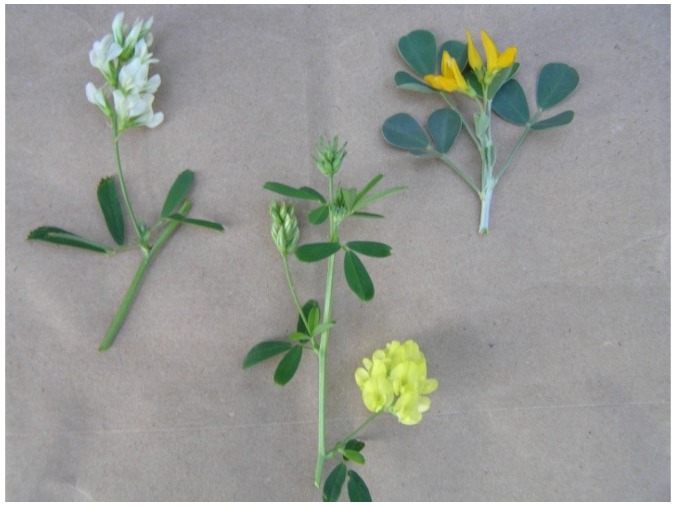
*Medicago sativa* clone M8 with cream flowers, left; hybrid, middle; *M. arborea* with yellow flowers, right. The figure also illustrates the long raceme on M8, left; the intermediate length raceme on hybrid, middle; and the short raceme on *M. arborea,* right.

The barrier was further weakened using clone M8, which has cream colored flowers (Figure 4). The M8 genotype has been crossed with *M. arborea* several times since its discovery, Table 2. It has always produced a hybrid, even when as few as 50 florets were pollinated. Hybrids of M8 × *M. arborea* are easily identified by yellow pigment in the flowers (Figure 4).

In total, M8 has produced 16 hybrids involving 1,350 florets pollinated. This is three-to-four-times more efficient in producing hybrids than MBms and WA 2071. In effect, M8 is a bridge-cross parent. Clone M8 was chosen for use as a seed parent, because it was male sterile and had cream-colored flowers. We did not know that it would become a bridge-cross parent in crosses, nor had we paid attention to its complex pedigree. 

The literature on the use of bridge-crosses contains several examples where the bridge-cross parent has a complex pedigree involving species crosses, often three species. In *Medicago*, trispecies bridge crosses were used to transfer *M. daghestanica* and *M. pironae* germ plasm into alfalfa [6]. Similarly, trispecies bridge crosses have been used for interspecific gene transfer in cotton [14], cucumber [15] and strawberry [16]. Interestingly, a three-way cross of *Drosophila* species overcame a fertility problem and permitted genetic analysis of reproductive isolation [17]. In our case, the bridge-cross parent involved three subspecies; however, all three were considered species until about 1975.

### 3.4. Argument that Hybridization Barrier Was Weakened by Selection of Seed Parents

The unsuccessful attempts to hybridize alfalfa and *M. arborea* have not been reported until now, except for a report in 1963 [5]. Our failed attempts prior to 1998 and 1998–2002, herein reported, used male sterile alfalfa seed parents that were unrelated to ones that have since produced hybrids. When hybrids were first obtained using seed parent MBms in 2003 in Wisconsin, and seed parent WA 2071 in 2007 in Queensland, seed parents 6-4ms and WA 2625, at respective locations, produced no hybrids under the same conditions and pollen sources. This supports the importance of the seed parent in the interspecific cross. Our failed attempts to produce the hybrid using *M. arborea* as the seed parent also support the importance of the alfalfa seed parent. 

In 2004, seed parent M8 was crossed with *M. arborea* in Wisconsin and produced three hybrids from only 300 crosses, while 6-4ms and MBms produced none. Clone M8 immediately became the seed parent of choice for the interspecific crosses for the next few years. Concurrently, 6-4ms and MBms also were used and were essentially checks on the efficiency of the M8 seed parent. Clone M8 consistently produced hybrids each time it was used, whereas the checks did not. This supports the notion that the hybridization barrier was lowered through selection of M8 as seed parent. 

### 3.5. Reproductive Abnormalities of Bridge-Cross Parent M8

Clone M8 was derived from a complex three-way × three-way cross involving subspecies sativa, coerulea and falcata, detailed in the Experimental Section. Subspecies parents were all normal in pollen production and seed set, whereas clone M8 has only a trace of pollen and functions as a male sterile in crosses. Lack of normal pollen production was a conspicuous reproductive abnormality. 

**Figure 5:**
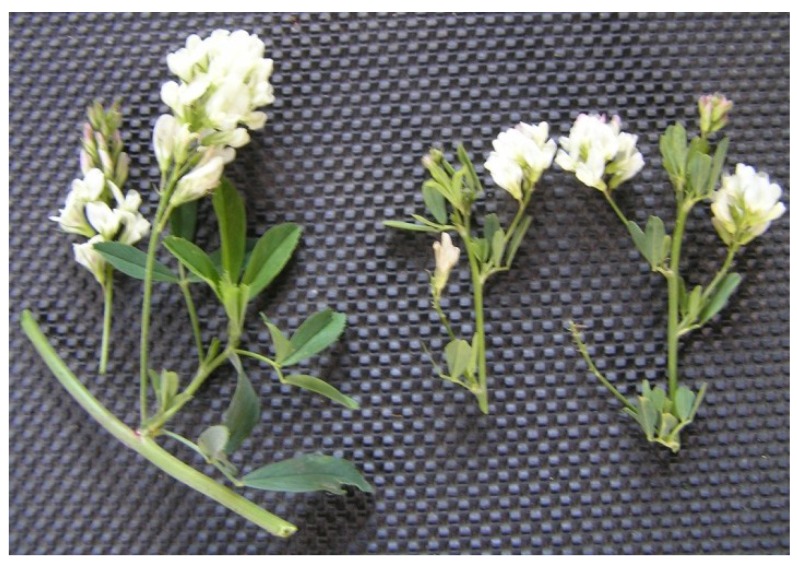
*Medicago sativa* clone M8, left, and two of its haploids, right. Haploids are smaller in every detail: small shoots, leaves and flowers; scant amount of small pollen and a few small seeds when crossed with other diploids or even fewer seeds when crossed with tetraploids.

The fact that M8 has cream flower color makes it easy to confirm self-progeny and reduced parthenotes (haploids). Preliminary identification of haploids is easy, because every morphological trait is half size (Figure 5). Six haploids of M8 have been confirmed over the years by flower color and chromosome counts. Parthenogenesis in M8 is considered another one of its reproductive abnormalities.

### 3.6. Experiments on Pollination Factors that Could Affect Hybrid Frequency of Clone M8

Clone M8 produces more hybrids with *M. arborea* than MBms and WA 2071. Furthermore, M8 has a trace of pollen and produces more self-progeny when hundreds of florets are self-pollinated. Could the extra pollen somehow be involved in recovering more hybrids from M8? Could it be as simple as retaining more pods with an interspecific hybrid embryo, when there is a normal seed from self-pollination also developing in the pod? The answer is no. Old records and recent work indicates most hybrids have come from single-seeded pods.

Could the trace of M8 pollen mentor *M. arborea* pollen? If so, additional M8 pollen could increase the hybrid frequency. An experiment where many M8 florets were tripped onto the pollination tool before adding *M. arborea* pollen produced 95 self-progeny and no hybrids. Again, the answer is no.

A similar experiment used M8 pollen collected from a large number of florets, and mixed with *M. arborea* pollen as above, to pollinate alfalfa clone 6-4ms. If a hybrid were produced on 6-4ms, it would indicate M8 pollen did something special in the pollination/fertilization process, because 6-4ms has not produced hybrids on its own. All 118 progeny were purple-flowered plants from 6-4ms × M8 crosses. No hybrids with *M. arborea* and no insights were obtained.

Finally, the behavior of the female gametophyte of M8 was examined in an experiment where pollen from *M. arborea* (yellow flowers) was mixed with pollen from alfalfa (purple flowers) by tripping equal numbers of flowers onto the same pollination tool. Cream flowered M8 was pollinated with the mixture. If an abnormality in the female gametophyte (embryo sac) allowed co-mingling of genetic material of two male parents, the resulting hybrid would have variegated flower color with co-expression of purple and yellow pigments. No hybrids with variegated flower color were found, nor were yellow-flowered hybrids with *M. arborea*. The *ca* 500 progeny were all purple-flowered hybrids with alfalfa. The result re-enforces the need for male sterile seed parents in making wide crosses. Fertilization involving genetic material from two male parents is not supposed to occur, and it was not detected here; but, there are such reports in the literature in both plants and animals [18].

Thus, these pollination factors did not appear involved in weakening the hybridization barrier. However, many other factors await study, beginning with gametogenesis and megaspore development, and continuing on to the physiology of reproduction. 

### 3.7. Weakening of the Barrier to Wide Hybridization and the Terms Genomic Disruption, Genome Shock and Hybrid Breakdown

These terms are metaphors used to describe abnormalities that have occurred after hybridization of diverse parents. Genomic disruption and genome shock explain events and processes, respectively, and hybrid breakdown includes all resulting abnormalities. The terms help explain the reproductive behavior of clone M8, given its complex subspecies parentage.

Barbara McClintock’s 1984 address to the Nobel Prize Foundation [19] outlines how the barrier to hybridization could be weakened. Titled: “The Significance of Responses of the Genome to Challenge”, the paper introduces the term “genome shock” and cites many types of challenges that can shock the genome, including species crosses. She further states: “In most known instances of such challenges, the types of response are not predictable in advance of initial observations on them”. In the case of M8, the response included reproductive abnormalities that may be involved in an unpredicted weakening of the interspecific crossing barrier. 

A plant patent by John Carman, 2004, available on the web [20], “Methods for Producing Apomictic Plants”, is based on crossing plants divergent in response to photoperiods and the timing of reproductive processes, including gametophyte development. The divergent plants often involve species and subspecies. In Carman’s model, apomixis sometimes occurs due to asynchronous expression of female developmental programs. Extensive literature supporting this is cited. Clone M8 was developed without knowledge of the patent, but it was bred using the same methods as outlined in the patent, and it produces a low frequency of progeny by apomixis. 

In the case of hybrid breakdown, the first generation of a cross involving diverse parents may be vigorous and fertile, but the F2 and later generations have reduced fitness for vigor and fertility. See Etterson *et al.* [21] for a review of this area, as well as discussion appropriate for autotetraploids. 

Clone M8 is a product of crossing divergent subspecies in two-way and three-way crosses and finally, a three-way × three-way cross. Thus, it was subjected to three generations of genome shock. In retrospect, reproductive abnormalities are no surprise. Considering how many genetically controlled processes could be affected by genome shock, it seems likely that several small interacting factors could cause imbalances in normal reproduction and that such imbalances could weaken the barrier to wide crosses. A full understanding awaits future research.

## 4. Conclusions

Alfalfa and *M. arborea* are reproductively isolated by a hybridization barrier. The barrier remained intact when *M. arborea* was the seed parent in crosses. The barrier was weakened by selection of cultivated alfalfa seed parents that produced a few hybrids after a large number of crosses. The barrier was further weakened by using a seed parent derived from complex crosses involving cultivated alfalfa and its subspecies relatives. This seed parent produced hybrids efficiently and serves as a bridge-cross parent to transfer genes from *M. arborea* to alfalfa. Bridge-cross parents in many species are products of crossing species and subspecies, as was the case in this study. The bridge-cross parent had reproductive abnormalities in pollen production and a low frequency of maternal haploids. However, these abnormalities could not be linked to lowering the hybridization barrier, in a limited number of experiments. 
